# Influence of Full Oral Feeding Acquisition on Growth of Premature Infants

**DOI:** 10.3389/fped.2022.928051

**Published:** 2022-07-14

**Authors:** Bibiana Chinea Jiménez, Silvia Ferrández Ferrández, Jesús Díez Sebastián, Miguel Saenz de Pipaon

**Affiliations:** ^1^La Paz University Hospital, Madrid, Spain; ^2^Department of Nursing, Universidad Complutense de Madrid, Madrid, Spain; ^3^Department of Pediatrics, Universidad Autónoma de Madrid, Madrid, Spain

**Keywords:** nasogastric feeding tube, premature infants, nurse, Neonatology, human milk

## Abstract

**Objective:**

The main objective was to describe the impact of full oral feeding achievement in very low birth weight infants on weight, length, and head circumference, measured as the change in *z*-score from 32 weeks to discharge, the time at which full oral feeding occurs.

**Methods:**

This was a longitudinal retrospective observational study on infants younger than 30 weeks of gestational age, admitted to the Neonatology Unit of La Paz University Hospital, Madrid (Spain), from January 1, 2019 to December 31, 2019. The infant's anthropometric characteristics (weight, height, and head circumference) were compared at birth, at 32, 34, and 36 weeks of gestational age, at the time of full oral feeding, and at discharge from the unit.

**Results:**

A total of 66 infants were included, gestational age at birth range from 24 to 30. Full oral intake occurred at 37.1 ± 2.1 weeks postmenstrual age (PMA). We found an inverse correlation between gestational age at birth and birth weight with PMA at which full oral feeding (FOF) is achieved. PMA at discharge was 38.6 ± 2.5 weeks. Age of full oral intake and discharge occurred later in infants who had patent ductus arteriosus, retinopathy of prematurity, and sepsis or received a blood transfusion. A positive correlation was found between days of oxygen and both parameters. However, we found no relationship between necrotizing enterocolitis or intraventricular hemorrhage with age at full oral feeding or age at discharge.

**Conclusions:**

The transition from gastric tube to oral intake did not affect growth. We found a close relationship between preterm infants birth, earlier younger than 30 weeks of gestational age, and low birth weight, with a delay in full oral feeding achievement that correlated with age at discharge.

## Introduction

Preterm infants have higher nutritional requirements per kilogram than term infants and are less tolerant of high fluid volumes (1). Physiologically stable preterm infants generally begin transition from tube feeding to oral feeding at 32–34 weeks' gestational age. This transition can take days or weeks (2). Achieving full oral feeding is an important step for preterm infants, given that it is an important discharge criterion indicating the maturity and health of the preterm infant (1).

Success in oral feeding depends on several factors: the infant's neurological and physiological maturity, the ability to concentrate on feeding, to organize oral-motor functioning, to coordinate swallowing with breathing, and to maintain physiological stability; as well as the ability of the caregiver to regulate the baby during feeding and to recognize and respond in a timely manner to the baby's behavioral and physiological signals, with the aim of preventing physiological decompensation and repeated stress. It is especially important to have a unit's consensus on nutrition/feeding and the nutritional protocol (2). Illness, may influence oral abilities. In our Unit, invasive, non-invasive ventilation, and CPAP-dependent infants remain fed by tube.

A retrospective review of exclusively breastfed preterm infants born weighing <1,800 g showed a decline in *z*-score for weight between birth and 35 weeks of 0.52, and another decline of 0.48 between 35 weeks and discharge (3). In this study, the transition was to breastfeeding and close to 80% were discharged home on breastfeeding, where direct breastfeeding was not fortified. The second decline in *z*-score for weight occurred when the transition between the nasogastric tube and oral feeding occurred.

## Justification

At the time of discharge, preterm infants present a decrease in growth, which could be explained by the transition from the nasogastric tube to oral intake, as can be inferred from the results of Marino et al. (3).

## Hypothesis and Objectives

We hypothesized that achievement of full oral feeding in very premature infants is associated with a decrease in growth. The primary aim was to describe the impact of achieving full oral feeding in very low birth weight infants on weight, length, and head circumference measured as the change in *z*-scores from 32 weeks to discharge, the time at which full oral feeding occurs.

### Secondary Aims

- To determine when the transition from gastric tube to exclusive oral nutrition occurs.
- To evaluate the effect of age at full oral feeding on:
  ° The length of hospital stay.
  ° The age at discharge.
- To analyze the effect of morbidity associated with prematurity with age at full oral feeding and length of stay or age at discharge.

## Methods

This was a longitudinal retrospective observational study on infants younger than 30 weeks of gestational age, admitted to the Neonatology Unit of La Paz University Hospital, Madrid (Spain), from January 1, 2019 to December 31, 2019.

### Sample

The study sample included all infants younger than 30 weeks of gestational age. Infants were excluded if they had congenital anomalies, genetic diseases, were admitted after 48 h of life, died in the first 7 days of life, were not receiving enteral nutrition, had incomplete medical records, or if at the time of discharge they were receiving gastric tube feeding.

Of the 98 preterm infants younger than 30 weeks of gestational age admitted during the study period, 7 died prior to discharge (5 in the first 7 days of life and 2 before de transition to full oral feeding, FOF) and 25 met the exclusion criteria.

The study, therefore, included 66 infants, with a range of gestational age from 24 to 30, mean ± standard deviation (SD) gestational age, and weight at birth of 28.3 ± 2 weeks and 1.1 ± 0.3 kg, respectively (see Table 1 for patient characteristics). In this cohort of preterm infants, parenteral nutrition lasted for a median of 8 days and lipid solution for 7 days. Approximately 63% of the volume of enteral nutrition consumed during the first 14 days of life was from own mother's milk (OMM) (Table 1).

**Table 1:** Demographic and anthropometric data of the newborns included in the study: Parenteral nutrition data during admission, enteral nutrition during the first 14 days of life, and fortification initiation and duration.

***N* = 66**	**Mean (SD)**
Gestational age	28.3 ± 2
Sex (*N*, %)	M 30 (45%), F 36 (55%)
Multiple	Yes 32 (48%), No 34 (51%)
Birth	Vaginal 14 (21%)
	Cesarean section 52 (78%)
Retarded intrauterine growth	Yes 9 (14%), No 57 (86%)
Birth weight (g)	1,104 ± 302
Birth weight (z-score)	0,005 ± 0,84
Length at birth (cm)	36,08 ± 3,15
Length at birth (z-score)	−0,30 ± 0,97
Head circumference at birth (cm)	25,34 ± 2,23
Head circumference at birth (z-score)	−0,31 ± 0,93
**Enteral nutrition during the first 14 days of life Median (Min, Max)**	
Volume OMM (ml)	752 (0–55130)
% OMM	63,16 (0–100)
Volume DHM (ml)	232 (0–2214)
% DHM	31,47 (0–100)
Volume premature formula milk (ml)	0 (0–536)
% Premature formula milk	0 (0–100)
Total volume (ml)	1239,0 (63–55138,0)
**Parenteral nutrition during the hospitalization Median (Min, Max)**	
Days of parenteral nutrition	8 (0–51)
Volume of parenteral nutrition (ml)	647 ± 434*
Days of lipids	7 (0–49)
Volume of lipids (ml)	89,90 ± 62,76*
**Human milk fortification**	
Age at fortification, days	38 (4–109)
PMA when fortification was started (weeks)	31 (26–33)
PMA when fortification was discontinued (weeks)	36 (27–89)

OMM, own mother's milk; DHM, donor human milk; PMA, postmenstrual age; M, male; F, female. *Mean (SD).

### Nutritional Protocol

International recommendations on early nutrition were followed, starting with parenteral nutrition immediately at birth. Enteral nutrition was introduced in the first 24 h after birth. Advances in enteral feeding volumes were made based on the signs of tolerance: vomiting, abdominal distension, and gastric residuals. The goal of enteral nutrition was to reach 150 ml/kg/day using OMM or donor human milk (DHM) when OMM was unavailable.

Donor human milk was available for infants born at <32 weeks or <1,500 g, at least during the first 3 or 4 weeks of postnatal age or until they reached 1,500 grams in weight, whichever occurred last. Once gestational age or the indicated weight was reached, if there was not enough OMM, the premature formula was administered for premature newborns. Standard fortification of human milk started when enteral feeding reached 100 ml/kg/day and continued until discharge.

Exclusive oral feeding is defined as the time of achieving full milk volume intake at the breast or by the bottle, for 24 h.

### Morbidity Definition and Measurement

Persistent ductus arteriosus (PDA) was only considered if treatment (pharmacological/surgical ligation) was reported as all hemodynamically significant PDA are considered for treatment in our protocol (4). Moderate-severe bronchopulmonary dysplasia diagnostic criteria were needed for oxygen at 36 weeks postmenstrual age (PMA) or discharge, whichever came first, and/or positive pressure ventilation or nasal continuous positive airway pressure at 36 weeks' PMA or discharge, whichever came first (5). The late-onset sepsis was defined as a positive blood culture obtained after 72 h of life (6). Intraventricular hemorrhage was defined as a Papile grade >2 (7). Infants underwent scheduled examinations and were graded according to the international classification of retinopathy of prematurity (8). Necrotising enterocolitis was defined as the presence of pneumatosis intestinalis with clinical signs and symptoms (9).

Weight, length, and head circumference measurements were taken at birth, at 32, 34, and 36 weeks of gestational age, at the time of full oral feeding, and at discharge. The *z*-score for each of these measurements was calculated based on the 2013 Fenton anthropometric neonatal charts (10).

### Statistical Method

The data were processed using a database in Microsoft Excel format, which was later imported for statistical treatment in the SAS program, version 9.4 (SAS Institute Inc. 2013; Base SAS 9.4 SAS/STAT–Statistical analysis; Cary, NC, USA). Statistically significant differences were those that presented a probability of error <5% (*p* < 0.05). First, a description of the sample included in the study was made.

As a pilot study, no power calculation analysis was performed. The infant's anthropometric characteristics (weight, height, and head circumference absolute values and *z*-scores) were compared at birth, at 32, 34, and 36 weeks of gestational age, at the time of full oral feeding, and at discharge from the unit.

### Descriptive Study

For the description of the continuous quantitative variables, the mean was used together with the SD in case of normality; otherwise, median and interquartile range were presented. The qualitative variables were described using absolute frequencies, and relative frequencies expressed as a percentage. For some variables, the BoxPlot graph representation was used.

### Analytical Study

Comparisons between continuous quantitative variables between independent groups were assessed mainly by parametric tests, using the Student's *t*-test when 2 groups were compared, or an analysis of variance (ANOVA) when the analysis involved 3 or more groups. When necessary, non-parametric tests were used, Kruskal–Wallis or Mann–Whitney *U* non-parametric tests. The frequency analysis between qualitative variables was performed using the chi-square test or Fisher's exact test when necessary.

The correlation analysis between the continuous quantitative variables was performed using Pearson's R correlation coefficient. The variables measured longitudinally (weight, length, and head circumference and their respective *z*-Score) were analyzed with repeated measures ANOVA, together with the Greenhouse–Geisser test. When the result was significant, the Bonferroni *post-hoc* test was used to explore all possible differences between two-to-two time points.

### Ethical Considerations

The study was approved by the Research Committee and by the Clinical Research Ethics Committee of La Paz University Hospital on January 13, 2020 and February 20, 2020, respectively. Ethics committee and approval number HULP: PI-4009.

## Results

At 32 weeks' PMA, we observed a decrease in weight, length, head circumference, and *z*-scores compared with birth (Table 2, *p* < 0.001). At 34 weeks' PMA, further decreases in weight and length *z*-scores were observed compared with 32 weeks (*p* = 0.005). No differences in head circumference *z*-scores were observed between these 2 periods (Table 2, *p* = 1.000).

**Table 2:** Evolution of growth and type of lactation during admission.

	**32 weeks' PMA**	**34 weeks' PMA**	**36 weeks' PMA**	**FOF Age** **Days of life** **61.7 ±25.3** **37.1 ±2.1 PMA**	**Discharge** **38,55 ±2,47 PMA**
Weight (gr)	1,346 ± 229	1689.9 ± 294.34	2070.69 ± 348.49	1956.4 ± 338.7	2649.17 ± 500.04
Weight z-score	−1.06 ± 0.60^a^	−1.22 ± 0.76^b^	−1.28 ± 0.96	−1.22 ± 0.98	−1.26 ± 0.83
Length (cm)	38.90 ± 2.30	40.78 ± 2.60	43.24 ± 2.34	42.60 ± 2.46	45.73 ± 2.51
Length z-score	−1.09 ± 0.86^a^	−1.41 ± 1.02^b^	−1.48 ± 0.98	−1.39 ± 1.02	−1.45 ± 1.00
Head circumference (cm)	27.48 ± 1.60	29.62 ± 1.54	31.60 ± 1.48	30.97 ± 1.58	33.46 ± 1.64
Head circumference (z-score)	−1.07 ± 1.02^a^	−0.90 ± 1.09^c.d^	−0.57 ± 1.05^c^	−0.66 ± 1.07	−0.28 ± 0.93^*d*^
Volume OMM (ml)^e^	353.60 ± 258.59	430.61 ± 326.29	400.47 ± 382.03		
% OMM	61 ± 38	53.74 ± 37.98	43.12 ± 40.43		
Volume DHM (ml)^e^	209.60 ± 212.96	195.64 ± 231.97	0 (0–945)		
% DHM	38 ± 38	27.36 ± 34.51	0 (0–100)		
Volume premature formula milk (ml)^*e*^	0 (0–300)*	0 (0–1,535)*	306 (0–1,080)		
% Premature formula milk	2 ± 10	19.50 ± 34.32	39.00 ± 19.04		
Volume infant formula (ml)^e^	0	0	0 (0–745)*		
% Infant formula	0	0	3,39 ± 16,86		
Volume hydrolysed formula (ml)^e^	0	0	0 (0–675)*		
% Hydrolysed formula	0	0	1,75 ± 13,25		
Total volume (ml)	577.68 ± 153.39	774.55 ± 227.55	861.86 ± 279.56		
Breastfeeding at discharge					Yes 33.8% No 66.2%

Data are expressed as mean ± SD for continuous variables, except *, which is expressed as median. ^a^Significant differences between birth and 32 weeks' PMA (P < 0.001). ^b^Significant differences between 32 weeks' PMA and 34 weeks' PMA (P < 0.005). ^c^Significant differences between 34 weeks' PMA and 36 weeks' PMA (P < 0.001). ^d^Significant differences between 34 weeks' PMA and discharge (P < 0.001). ^e^During three consecutive days. FOF, full oral feeding; PMA, postmenstrual age.

At 36 weeks' PMA and at discharge, no differences in weight or length z-scores were found compared with 34 weeks' PMA (Table 2, *p* = 1.000). A significant increase in the head circumference *z*-score was observed at 36 weeks' PMA and at discharge compared with 34 weeks' PMA (Table 2, *p* < 0.001).

**Figure 1:**
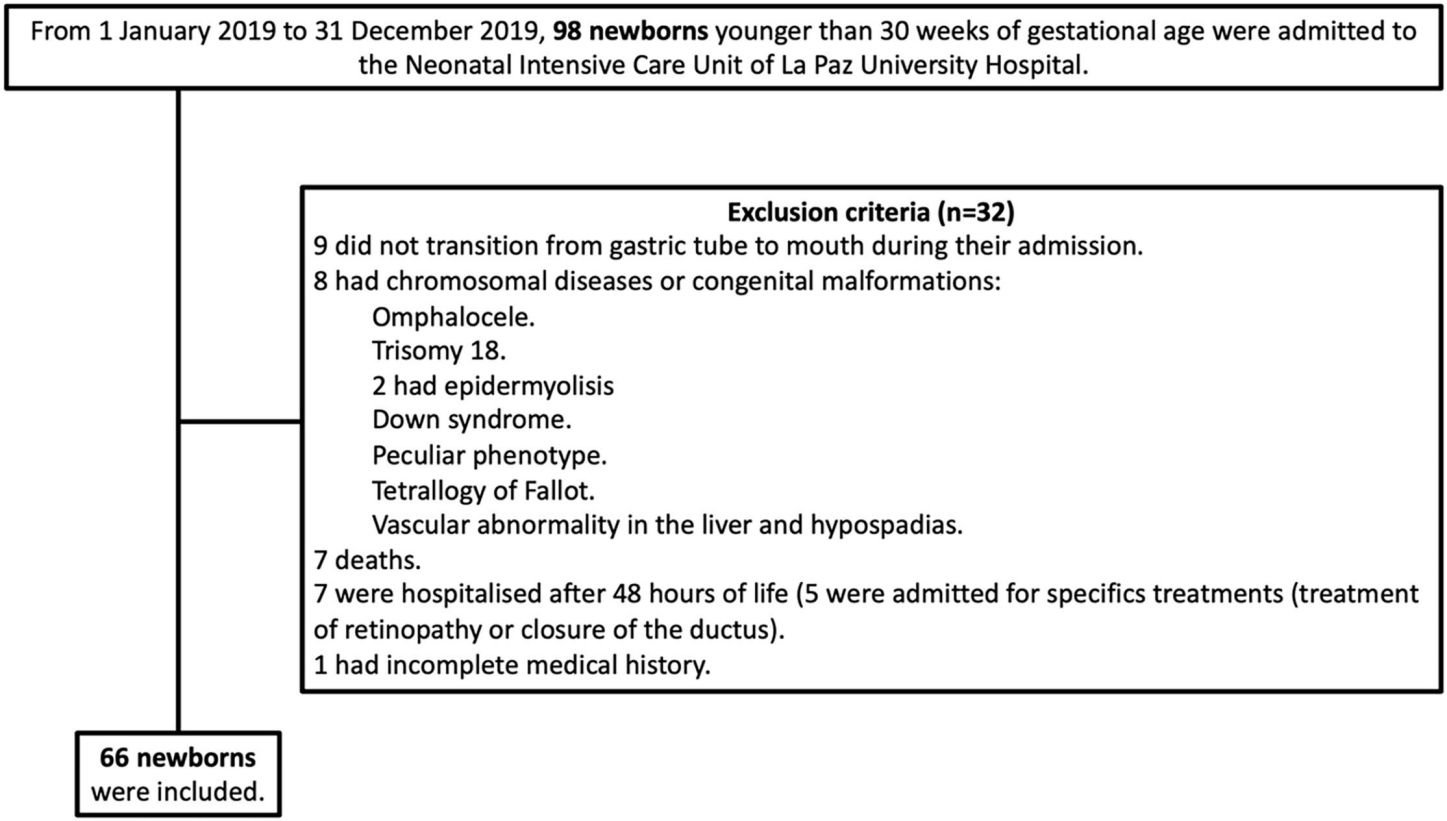
Flowchart of included and excluded newborns.

The full oral intake occurred at 37.1 ± 2.1 weeks' PMA. We found an inverse correlation between gestational age at birth and PMA at which full oral feeding was achieved (*p* < 0.001, Figure 1). There was also an inverse correlation between birth weight and PMA at which full oral feeding was achieved (*p* < 0.001, Figure 1).

**Figure 2:**
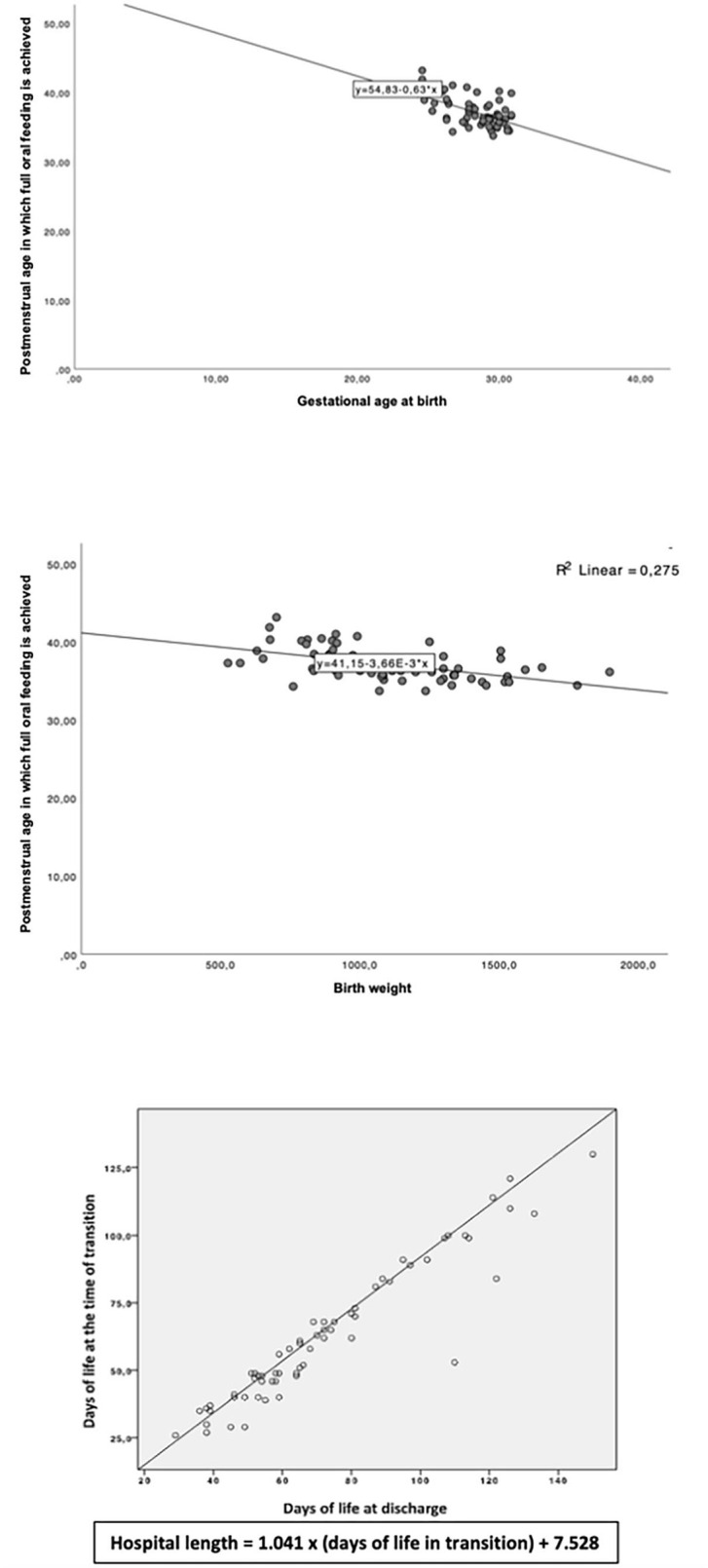
Relationship between gestational age at birth and postmenstrual age at which full oral feeding was achieved; Relationship between birth weight and postmenstrual age at which full oral feeding was achieved; relationship between age at discharge and age at full oral feeding.

The PMA at discharge was 38.6 ± 2.5 weeks. There was a correlation between age at which full oral intake occurred and age at discharge. Hospital length (HL) was also related to full oral feeding age. HL (days) = 1.041 × (days of full oral feeding) + 7.528 (Figure 2).

The OMM consumption during hospitalization decreased over time. The percentage of OMM intake relative to total enteral intake was 63, 64, 57.5, and 24% during the first 14 days and at 32 weeks' PMA, 34 weeks' PMA, and 36 weeks' PMA, respectively. Before 32 weeks' PMA, OMM intakes are completed with DHM, depending on the patient's weight (it is maintained until reaching 1,500 g) and the availability of DHM in the milk bank. From 32 weeks' PMA, feedings were completed with premature formula. At the time of discharge, only 33.8% had managed to establish breastfeeding directly at the breast (Table 2).

We found a correlation between days of oxygen, the presence of PDA, retinopathy of prematurity, sepsis, and transfusion, and age at full oral feeding and age at discharge. However, we found no relationship between necrotizing enterocolitis or intraventricular hemorrhage with age at full oral feeding and age at discharge (Table 3).

**Table 3:** Associations between Pretermassociated morbidities and age at full oral feeding and age at discharge.

	**PDA yes (34)**	**PDA no (32)**	***P*-value**
Full oral intake	72.60 ± 25.2	50.0 ± 19.8	0.000
Length of stay	85.0± 28.0	57.5 ± 19.2	0.000
	**PDA Med yes (22)**	**PDA Med no (44)**	
Full oral intake	80.64 ± 26.56	52.18 ± 18.59	0.000
Stay	94.77 ± 28.81	60.16 ± 18.51	0.000
	**PDA surgery yes (7)**	**PDA surgery no (59)**	
Full oral intake	91.57 ± 23.67	58.12 ± 23.19	0.001
Stay	103.71 ± 24.94	67.90 ± 25.59	0.001
	**NEC Yes (1)**	**NEC No (65)**	
Full oral intake	100	61.08 ± 25.02	0.128
Stay	113 ± 25.02	71.06 ± 27.39	0.134
	**NEC Qx yes (1)**	**NEC Qx no (65)**	
Full oral intake	100	61.08 ± 25.02	0.128
Stay	113 ± 25.02	71.06 ± 27.39	0.134
	**IVH yes (22)**	**IVH no (44)**	
Full oral intake	69.09 ± 24.27	50.0 ± 19.8	0.192
Stay	85.0± 28.0	57.5 ± 19.2	0.072
	**ROP yes (22)**	**ROP no (44)**	
Full oral intake	86.77 ± 21.41	49.11 ± 16.08	0.00
Stay	99.45 ± 24.20	57.82 ± 16.71	0.00
	**Sepsis yes (25)**	**Sepsis no (41)**	
Full oral intake	80.20 ± 25.52	50.37 ± 25.02	0.00
Stay	91.72 ± 27.92	59.49 ± 19.30	0.00
	**Transfusion yes (28)**	**Transfusion no (38)**	
Full oral intake	81.82 ± 23.84	46.82 ± 13.20	0.00
Stay	93.96 ± 26.66	55.29 ± 13.19	0.00

*Mean and standard deviation. Data are expressed as days*.

## Discussion

This study aimed to evaluate the effect of the transition to oral feedings on growth and age at discharge. We did not find a further decrease in weight or length *z*-scores from 34 weeks' gestation to discharge, when full oral feeding occurred. The weight and length *z*-scores decreased from birth to 34 weeks' PMA. The head circumference decreased from birth to 32 weeks' PMA. The head circumference *z*-score increased from 32 weeks to discharge, and the weight and length *z*-scores remained stable from 34 weeks' PMA to discharge.

The weight gain is maybe influenced by oral feeding. Full oral feeding was achieved after 34 weeks' PMA and before discharge. The present study results showed no changes in the weight or length *z*-score from 34 weeks' PMA to discharge, weight, and length gain continue during the transition phase, from gavage to oral, without slowing down, and showed an increase in the head circumference *z*-score, in head circumference growth. A possible explanation of our findings is that full oral feeding was achieved by bottle, not at the breast, in 66% of the infants. The milk volume administered did not change between before and after achieving this goal. In our study infants were followed for 5 weeks.

These results are consistent with those reported by Lehnart Vargas et al. (11), with a GA at birth of 33,6 (±1.5) weeks, although the infants included in our study were more immature. These researchers did not find significant differences in growth, represented by weight gain, and concluded that their growth was not affected by the level of oral skill. PMA at which full oral intake was attained, between 36 and 38 weeks, was similar to that reported in the present study. However, Marino et al. (3) found a decrease in the weight and length *z*-scores at 35 weeks of gestational age and at discharge, but not in head circumference, with a population similar to the one reported here, with a percentage of breastfeeding at the discharge of 78.1%.

In our results, full oral intake occurred at a median PMA of 36.6 (33.7–43.1) weeks. These results are similar to those reported by Khan et al. (12) in which they found full oral feeding skills were reached at 37.1 (35.6–38.4) weeks in extremely preterm infants and at 34.7 (34.3–35.6) weeks in very preterm infants (*p* < 0.001) Kangaroo mother care was promoted in our study, (13) but other interventions that could further improve oral capabilities were not applied, such as prefeeding oral stimulation and the use of a checklist, as described by da Rosa Pereira et al. (14).

Our findings confirmed that gestational age at birth influenced the development of full oral feeding, preterm neonates who were less mature at birth were more mature at attainment of independent suckle-feeding. It is acknowledged that extremely preterm infants have immature motor skills and lack coordinated sucking ability. This result is in accordance with the results of Khan et al. (12) and Jackson et al. (15). Birth weight was associated with the postmenstrual age at the time of achievement of full enteral feeding, and Jackson's report was related to the full oral intake with the weight at birth. Thus, we can confirm that premature infants born with a lower gestational age and lower birth weight take longer to reach full oral feeding (12, 15).

Preterm infants must achieve independent oral feeding before discharge. Achievement of independent oral feeding remains the most common barrier to discharge in preterm infants. The timing at which full oral feeding is attained is associated with the age at hospital discharge and may reduce associated hospital costs. Our results can thus help predict when patients will be discharged: once they have achieved full oral feeding, it will be 1 week after achieving this milestone. The next step is to evaluate prospectively whether a practice change to earlier attainment of full oral feeding enables earlier hospital discharge. Finally, we found several morbidities related to prematurity that delay the goal of achieving full oral feeding, especially those related to the use of oxygen- and PDA-treated infants. However, we found no association between full oral feeding and necrotizing enterocolitis or intraventricular hemorrhage, probably because a larger sample size would be necessary (15). Few studies have investigated the effect of co-morbidities on the achievement of independent oral skills. Preterm infants have difficulty establishing oral feeding skills because their cardio-respiratory systems are functionally immature. The use of oxygen possibly because it can disrupt the individual rhythms of sucking, swallowing, and breathing, which are critical for achieving coordinated suckling. PDA is a common problem in preterm infants. PDA further limits the infant's capacity to progress to oral feeding competence. The prolonged patency of ductus arteriosus is often related to an increased hospital morbidity, oxygen dependence, and subsequent BPD.

### Limitations

The main limitation of this study was its retrospective design. Other limitations: Information on mechanical ventilation was not collected; we did not have into account when the transition from oral tube to oral intake begins and the duration, only when it was achieved; no measurement tool was used for the evaluation of readiness for feeding and it was conducted only in one Center.

## Conclusion

In our study, in which only one third of the infants established breastfeeding at discharge, two thirds were bottle fed, transition from gastric tube to full oral feeding does not affect growth. We found a relation between preterm birth, earlier than 30 weeks gestation and low weight at birth with a delay in full oral feeding achievement that correlated with age at discharge.

## Data Availability Statement

The original contributions presented in the study are included in the article/supplementary material, further inquiries can be directed to the corresponding author.

## Ethics Statement

The studies involving human participants were reviewed and approved by Comité de Ética de la Investigación con Medicamentos del Hospital Universitario La Paz. Written informed consent to participate in this study was provided by the participants' legal guardian/next of kin.

## Author Contributions

The planning, conception, and design of the study were proposed by BC together with MS. Data collection was carried out by SF and BC. The statistical analysis was performed between JD, MS, and BC. And finally, the conclusions and writing of the article were carried out by BC and MS, with the critical review of SF and JD. All authors contributed to the article and approved the submitted version.

## Funding

This work was funded by the Fellowship 2021-2023 ESPGHAN Networking Grant 2021 to MS (PI).

## Conflict of Interest

The authors declare that the research was conducted in the absence of any commercial or financial relationships that could be construed as a potential conflict of interest.

## Publisher's Note

All claims expressed in this article are solely those of the authors and do not necessarily represent those of their affiliated organizations, or those of the publisher, the editors and the reviewers. Any product that may be evaluated in this article, or claim that may be made by its manufacturer, is not guaranteed or endorsed by the publisher.
